# Supplementary material for: A mutation-level covariate model for mutational signatures
Source: PLoS Comput Biol. 2023 Jun 5;19(6):e1011195. doi: 10.1371/journal.pcbi.1011195 (PMC10270581; doi:10.1371/journal.pcbi.1011195)
Supplement: S1 Text — Fig A. Radar plots showing reconstruction of MCSM model parameters using generated data. The angular axis is the signature number and the radial axis is its respective parameter value. We show that our method is able to recover the parameters in five different tests, and provide also the parameters estimated by LDA. Fig B. Radar plots showing reconstruction of JMCSM model parameters using generated data. Since we learn the strand bias per signature, we won’t necessarily reconstruct the exact parameters. However, the ratio between the normalized parameters that indicates the bias of the signatures and was used in Fig 5, should be preserved instead. The radial axis indicates the ratio between the normalized parameters and the angular axis indicates the signature. We show that our method is able to reconstruct the bias trends in most cases, across five different tests. Table A. A comparison between the held-out log-likelihoods achieved by JMCSM and gLDA on generated data based on BRCA across five different tests. In all cases, JMCSM yields better results. Table B. A comparison between strand-sensitive and strand-oblivious models using genomic strand as a mutation-level feature. It is evident that in all instances that JMCSM and MCSM yield lower likelihoods than their strand indifferent variants. Table C. A summary of the number of iterations the algorithms ran on generated data before convergence, across five different tests. In all cases, the held-out log-likelihood achieved convergence before the 25th iteration. Table D. A summary of the number of iterations the algorithms ran on real data before convergence in all but one instance (when gLDA was executed on BRCA with the replication strand as a feature, it took 29 iterations). Although we report the mean of the held-out likelihood of the last 25 iterations, it doesn’t have any effect on the results or the conclusions of the paper since the variations in the held-out loglikelihood are negligible relatively to the dif [file pcbi.1011195.s001.docx]

A mutation-level covariate model for mutational signatures

Supplementary information

Itay Kahane, Mark DM Leiserson and Roded Sharan

Parameter reconstruction using generated data

We tested our code on generated data to validate it. The data was generated by the following process:

1. For MCSM we drew Dirichlet parameters (a' and b') from a standard normal distribution with $\sigma=1$ and $\mu=0$, and generated the mutation data using the generative process described in the text. We did it with different signatures, and number of samples (and their sizes) with respect to the original databases.
2. For JMCSM we used a similar process, and then we multiplied $a=exp(a^{'})$ and $b=exp(b^{'})$ by $G=1000$. Note that this is necessary in the JMCSM framework, as it is assumed that each strand's exposure is close to the inherent exposure.

Then, the data was split to a train set and a test set using the same process described in the text.

MCSM:

We provide the parameters reconstructed using MCSM and LDA across 5 different sets of generated data. The parameters are given in radar plots, where the angular axis is the signature number and the radial axis is its respective parameter value. The black and the orange dashed lines indicate the $a$ and $b$ parameters of the generated data, the yellow and the green dashed lines indicate the parameters $a$ and $b$ estimated using MCSM, and the blue line indicates the $a$ parameters estimated using LDA. The results are given in Figure A.

Figure A


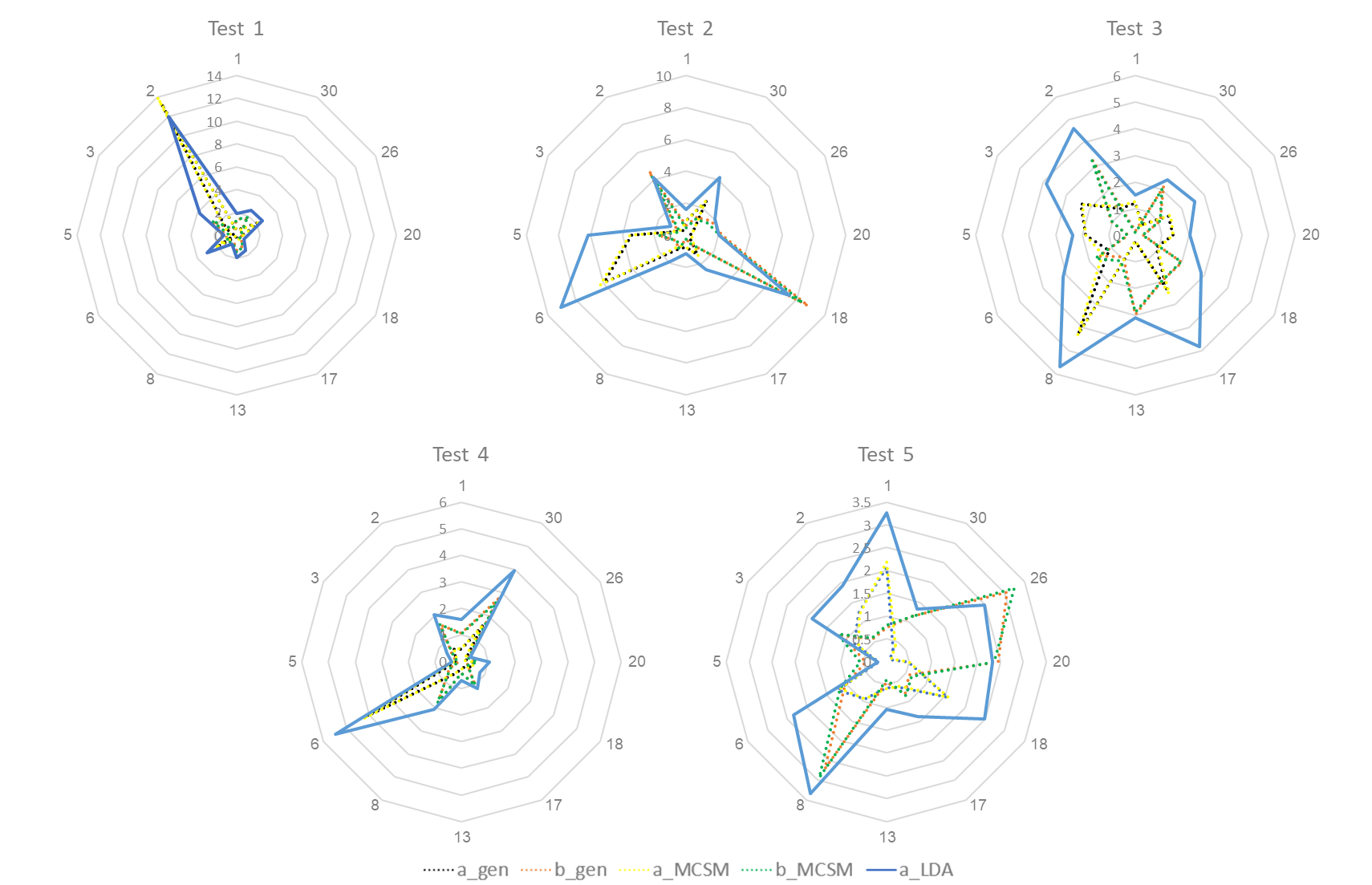


It can be seen that MCSM is able to reconstruct the model parameters well while LDA captures characteristics from both vectors. However, in some cases LDA is good enough to get similar or slightly better held-out log-likelihoods than MCSM (this happened in tests 4 and 5). Note that this coincided with the results in the paper.

JMCSM:

Since we learn the strand bias per signature, we won't necessarily reconstruct the exact parameters. However, the ratio between the normalized parameters $r_{i}=\frac{a_{i}}{\sum_{k=1}^{K} a_{k}}/\frac{b_{i}}{\sum_{k=1}^{K} b_{k}}$ , that indicates the bias of the signatures and was used in figure 1, should be preserved instead. The radial axis indicates $r_{i}$ and the angular axis indicates the signature. The results are given in Figure B (the values of the generated data and of the estimated parameters using JMCSM are in blue and orange respectively.

Figure B


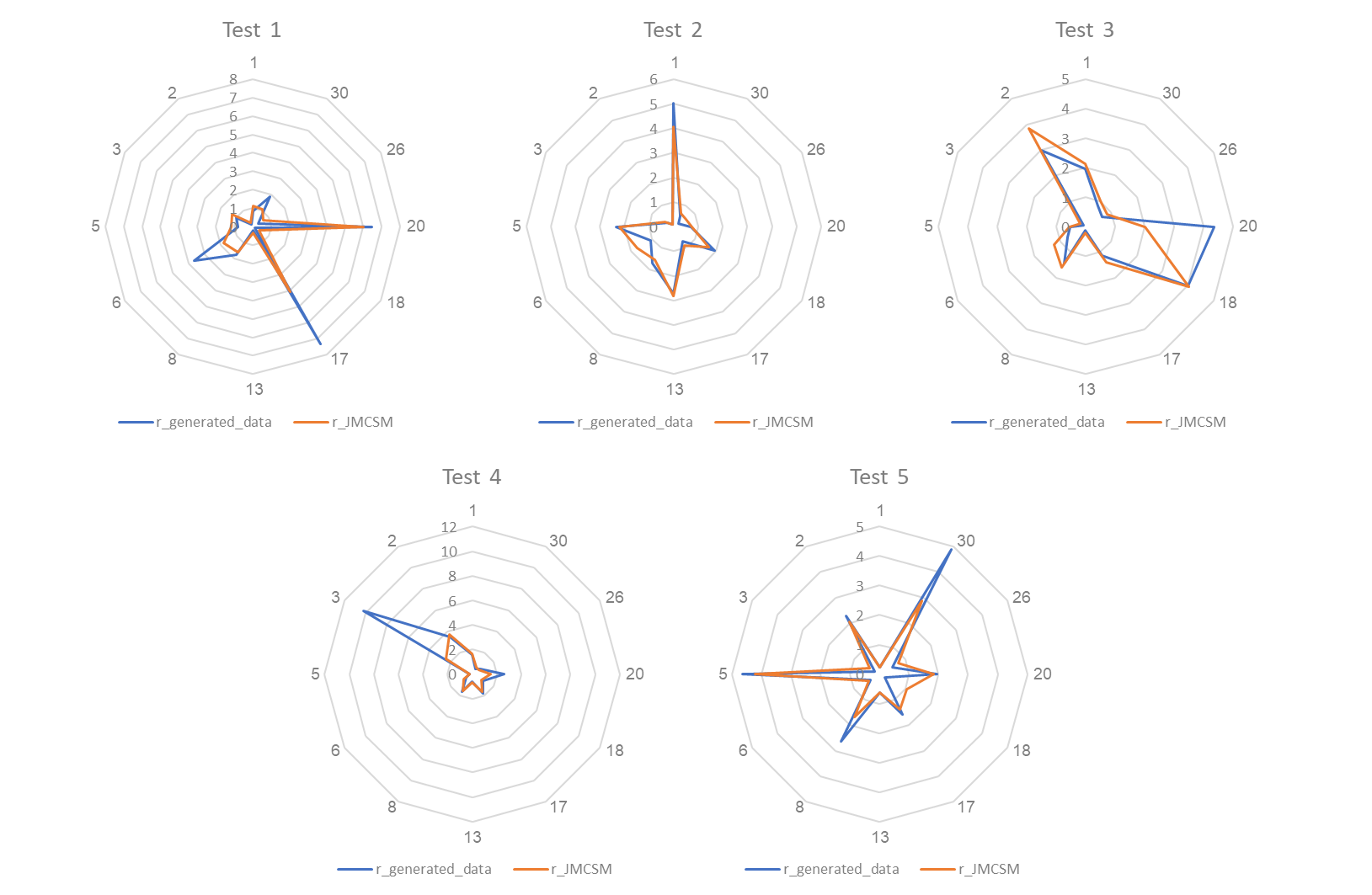


It is evident that JMCSM is able to reconstruct the bias trends in most cases.

For example, in test 1 the algorithm recognized that signatures 17 and 20 had the highest biases towards the strand that was drawn with parameters $a$ in test 1, and that signatures 13, 18, 26, 30, have small $r_{i}$. However, it didn’t calculate the bias of signature 6 correctly. Similar results can be seen in all other tests.

In Table A we present the held-out log-likelihood calculated using JMCSM and gLDA for each one of the 5 tests we ran.

Table A

Here, JMCSM provide better log-likelihoods than gLDA in all 5 tests.

Genomic strand indifference

In the main paper we state that we couldn't find an advantage when using our models with the genomic strand identity of the pyrimidine base as a feature. To do so, we performed a similar test presented in Table 4, with the genomic strand feature (as stated above) instead of replication strand, while tossing mutations with no information on the replication strand.

The results are given in Table B.

Table B

|  | JMCSM | gLDA | %diff | MCSM | LDA | %diff |
| --- | --- | --- | --- | --- | --- | --- |
| BRCA | -1996644 | -1996589 | 0.003% | -2218888 | -2215837 | 0.138% |
| MALY | -694977 | -694937 | 0.006% | -718582 | -718491 | 0.013% |
| CLLE | -111878 | -111866 | 0.010% | -112708 | -112703 | 0.004% |

It is evident that in all instances that JMCSM and MCSM yield lower likelihoods than their strand indifferent variants.

Held-out Likelihood Convergence

As stated in the paper, our convergence criterion for the stochastic EM algorithms was local maximum attaining while we still ran our algorithms for 50 iterations for robustness.

In tables C and D we summarize the number of iterations the algorithms ran on generated and real data respectively before convergence.

Table C

| Dataset | Test no. | JMCSM | gLDA | MCSM | LDA |
| --- | --- | --- | --- | --- | --- |
| BRCA | 1 | 25 | 16 | 6 | 16 |
|  | 2 | 23 | 13 | 9 | 16 |
|  | 3 | 21 | 13 | 4 | 11 |
|  | 4 | 22 | 15 | 9 | 14 |
|  | 5 | 24 | 14 | 4 | 11 |
| MALY | 1 | 11 | 8 | 2 | 5 |
|  | 2 | 14 | 10 | 3 | 9 |
|  | 3 | 15 | 11 | 6 | 4 |
|  | 4 | 15 | 11 | 4 | 3 |
|  | 5 | 11 | 8 | 3 | 5 |
| CLLE | 1 | 8 | 8 | 3 | 8 |
|  | 2 | 12 | 8 | 2 | 7 |
|  | 3 | 5 | 7 | 5 | 6 |
|  | 4 | 11 | 9 | 2 | 4 |
|  | 5 | 11 | 9 | 4 | 6 |

Table D

| Strand | Dataset | JMCSM | gLDA | MCSM | LDA |
| --- | --- | --- | --- | --- | --- |
| Replication | BRCA | 23 | 29 | 9 | 4 |
|  | MALY | 17 | 10 | 6 | 4 |
|  | CLLE | 9 | 13 | 7 | 9 |
| Genomic | BRCA | 16 | 19 | 5 | 7 |
|  | MALY | 15 | 10 | 4 | 5 |
|  | CLLE | 17 | 13 | 5 | 5 |

In all the tests that were done on generated data, the algorithms converged well before the 50^th^ iteration, and that the latest convergence was achieved after 25 iterations.

When executed on real data, the algorithms converged before the 25^th^ iteration in all but one instance (when gLDA was executed on BRCA with the replication strand as a feature, it took 29 iterations). Although we report the mean of the held-out likelihood of the last 25 iterations, it doesn't have any effect on the results or the conclusions of the paper since the variations in the held-out loglikelihood are negligible relatively to the difference between the held-out loglikelihood achieved by JMCSM and gLDA.
