# Supplementary material for: A mutation-level covariate model for mutational signatures
Source: PLoS Comput Biol. 2023 Jun 5;19(6):e1011195. doi: 10.1371/journal.pcbi.1011195 (PMC10270581; doi:10.1371/journal.pcbi.1011195)
Supplement: Attachment — Submitted filename: 2nd Review Answers V3.pdf [file pcbi.1011195.s002.pdf]

Reviewer 1

Kahane et al. present a Latent Dirichlet Allocation model in which they incorporate covariates to observed categorised mutations in order to extract mutational signatures. Statistical methods for signature analysis are still lagging, so this work can be useful for linking signatures to their aetiology, using signatures in order to specify patients, etc. The example they use of covariates is that of strand-specificity. Strand biases have already been assessed in COSMIC signatures, and are even already available in the COSMIC website (e.g. https://cancer.sanger.ac.uk/signatures/sbs/sbs2/). The authors should emphasize more strongly what is novel in their work.

The paper presents novel models that take mutation-level covariates into account, we have now emphasized the novel aspects in the Introduction.

The link to the covariates is done on parameter α', i.e. the log-transformed Dirichlet parameter, and as such it determines both mean and variance of signature exposures, which should be made clearer.

We have now clarified the way the covariate affect the model (p. 4).

The interpretation of results is on the normalised Dirichlet parameter α/sum α_i, and is compositional data. Therefore, signature-specific results are difficult and this needs to be made clearer in Figures 5 and 6.

We have now explained more clearly the caveats of the parameter ratio approach in Figure 5 and how it is addressed in the results of Figure 6 to pinpoint the relevant biased signatures (p. 10).

The paper the authors claim to present a way of extracting signatures and exposures (e.g. Conclusions: We have developed new topic models to account for mutation-level covariates when learning mutational signatures and their exposures) However, they assume that mutational signatures are known and only estimate the exposures - which needs to be made clearer throughout the paper.

We have stated this point (of known signatures) more clearly (p. 3,11).

α' is not defined in text.

We added an explicit definition of the a_i and a' parameters (p. 3)

I did not understand the held-out log-likelihood comparison method of assessing performance of the competing methods, please expand the explanation.

We have clarified this test procedure (p. 7).

SBS2, SBS13 and SBS26 are put forward as signatures with strand bias. SBS13 does not appear to have strand bias according to https://cancer.sanger.ac.uk/signatures/sbs/sbs13. Please discuss.

We now cite the evidence for the replicative strand bias of SBS13 (" The topography of mutational processes in breast cancer genomes", Table 1, row 4).

Reviewer 2

This manuscript proposes two novel topic models for mutational signature learning. Unlike previous methods, these models explicitly account for the effect of covariates on the exposures to different signatures. The authors apply their methods to real data and conclude that they obtain a better fit to held-out data and that they identify known strand-specific biases, and find new ones.

We have several major concerns, one being that there are no simulation studies that prove that the proposed models and learning algorithms do what they set out to do. Additionally, the text is at times difficult to follow. We detail our concerns below.

We address the specific comments in detail below.

The model description is unclear: what is a and b, and what is z drawn from? The symbols are introduced in the plate models before they are explicitly defined in the text, which makes this a very unpleasant read. In Figure 1, what is z drawn from? Is it a multinomial distribution with Nt and \theta as parameters? And how does it generate m? And why is a_k generated from a Gaussian distribution?

The symbols a, b are defined before the first plate model they appear in, z is now explained (p. 3). The generation process here is LDA, so z is drawn as the referee guessed. We have clarified the text accordingly (p. 3). a'_k is drawn from gaussian distribution as in LDA. Regarding a'_k, we followed [20].

In figures 1 and 2: The node for \gamma is greyed out as an observed quantity, which seems odd as it is defined in the text as the probability to draw a mutation m from signature k.

Correct. We specified in the text that we assume that the signatures themselves are known. We have now clarified this assumption (p. 3).

In figures 3 and 4: The node for the inherent exposures is white, indicating it is an unobserved quantity. Later in the text, we see that it is inferred separately using a non-joint model, so in the scope of the joint model, it is given and so should be greyed out as an observed quantity.

We followed this recommendation and clarified the special status of the exposures in the legend and text.

Page 5: “we also define a guided LDA (gLDA) model variant which is fed with external information on the exposures as in JMCSM”. In the plate model this is an unshaded node, which means it is unobserved. But in the text, it is referred to as external information, which is confusing. Which is it?

Fixed, see answer to the previous comment.

In model learning, the authors should clarify what is the objective function. Are they computing the maximum marginal likelihood? If so, which parameters do they marginalize over and which do they optimize? And how are they including the prior information in the estimation? Similarly, it should be explained why model comparison based on the held-out log-likelihood is valid for the particular marginalization applied.

We now clarify that we marginalize over all multinomial parameters draws from the Dirichlet distributions. Since we deal with log-likelihood, the prior parameter sigma appears as an additive constant that doesn't affect the learning process, so it was omitted in equation (6). We clarified that in the text (p. 7). Likelihood on held-out data is a standard way for comparing models with different parameterizations.

Additionally, the authors must describe their learning algorithms in more detail so that readers are able to understand the procedure and replicate it.

We have detailed the learning algorithm (p. 6)

Page 6: “we run SEM for 50 iterations”. Since this is an EM algorithm, can’t the authors assess the convergence of the objective function directly?

To show convergence, we present the incremental difference in held-out log likelihood of the first 25 iterations (starting from iteration #2 minus iteration #1) in the following table.

	JMCSM			MCSM			gLDA			LDA		
#	BRCA	MALY	CLLE	BRCA	MALY	CLLE	BRCA	MALY	CLLE	BRCA	MALY	CLLE
1	107317	36338	2967	148809	89222	7164	44739	39201	204	161038	38114	21330
2	31375	7920	994	36377	5044	1117	12899	3345	77	28726	2601	832
3	10400	1958	319	6294	746	236	3950	541	64	2758	575	117
4	4288	682	120	103	242	49	1728	186	28	-725	-248	95
5	1948	317	54	2252	65	26	706	76	11	737	469	3
6	1192	122	27	540	-94	43	351	30	9	-1425	9	26
7	633	67	17	1969	39	-47	158	4	4	730	17	13
8	374	26	15	675	216	51	140	4	-1	-975	-311	35
9	244	42	-1	-1350	-3	-5	55	2	-2	-519	-42	-26
10	215	10	9	1579	-68	-27	20	-6	-1	921	9	-2
11	140	20	-1	-1017	208	-12	8	-2	5	1659	116	-20
12	81	4	5	702	-115	-8	5	2	2	-2229	40	-13
13	43	8	6	1158	210	-5	0	-1	0	213	218	31
14	61	9	-6	781	-340	16	9	-1	0	1082	-550	3
15	44	3	4	-1211	-78	4	-2	1	-5	-1522	0	-15
16	16	5	-4	-702	105	-8	2	-3	-2	417	99	9
17	15	-10	3	-1066	295	-7	5	-1	1	-610	174	34
18	8	6	-5	1677	168	-45	1	-4	1	2532	-136	-35
19	29	9	8	-675	-269	-17	-8	-2	2	-1597	584	-1
20	7	5	-3	-359	-1	32	7	5	-2	-1502	-591	15
21	10	-1	3	-93	-167	8	-15	0	2	224	200	24
22	-9	7	2	117	79	3	7	5	0	864	110	-6
23	-5	-6	4	594	49	17	7	2	1	540	-33	13
24	6	-8	-7	1423	-44	20	-7	0	-6	-574	84	-13
25	-4	-5	3	-1534	-43	11	-1	2	3	317	76	-9

One can see that our main models, JMCSM and gLDA converge after 10-20 iterations depending on the database and yields almost noise-free results. This is a result of the fact that the concentration parameters we get in this model are very high, resulting in an almost deterministic Dirichlet distribution (from which the exposures are drawn).

Our less refined models, MCSM and LDA stop improving faster, and yield much noisier results. However, despite being noisy, when testing on MALY and CLLE, MCSM yields better results than LDA almost consistently. In the following table, we present the likelihood difference between MCSM and LDA (MCSM - LDA) in the last 25 iterations for all 3 databases:

	MCSM - LDA		
#	BRCA	MALY	CLLE
26	-888	1186	-42
27	752	854	-50
28	1044	882	-68
29	-743	959	-19
30	-1243	657	51
31	-948	491	49
32	580	-33	28
33	301	464	-1
34	-109	429	-24
35	1156	150	20
36	-22	283	11
37	-91	-220	28
38	644	198	27
39	326	478	94
40	-1605	836	144
41	-1354	900	74
42	-962	466	70
43	-933	640	14
44	-2365	517	60
45	-1687	331	-13
46	-760	520	41
47	-90	345	42
48	-829	548	51
49	-1395	621	70
50	-1912	525	53

Before applying their models to real data, the authors must benchmark them on simulated data, in order to see whether their learning method is working and whether they actually recover ground truth strand biases. Without such assessment, it is difficult to trust the results on real data.

We ran simulations as requested and describe the results below, however since the proposed simulation could be artificial and not representative of real data we chose to exclude them from the paper. We note that this comment appeared already in our point-by-point response to the RECOMB reviews in the original PLOS submission.

To generate data for MCSM, we drew Dirichlet parameters (a' and b') from a standard normal distribution with $\sigma = 1$ and $\mu = 0$, and generated the mutation data using the generative process described in the text. We did it with different signatures, and number of samples (and their sizes) with respect to the original databases.

In the following examples, a_gen and b_gen are the Dirichlet parameters used to generate the data (where a_gen = exp(a') and b_gen = exp(b')), while a_est and b_est are the results of the reconstruction process.

To demonstrate the ability of our MCSM code to reconstruct the parameters, we followed the process described above, and generated 5 independent databases based on BRCA.

The generated Dirichlet parameters and their estimations are reported below (In the following examples, a_gen and b_gen are the Dirichlet parameters used to generate the data (where a_gen = exp(a') and b_gen = exp(b')), while a_est and b_est are the results of the reconstruction process):

MCSM Test 1:

Sig	1	2	3	5	6	8	13	17	18	20	26	30
a_gen	1.54	0.44	0.75	1.29	2.38	0.37	1.00	2.95	0.84	0.59	0.14	0.70
a_est	1.59	0.39	0.70	1.19	2.02	0.37	0.91	2.87	0.78	0.60	0.19	0.65
b_gen	0.50	1.55	1.31	1.41	3.01	1.39	3.99	1.27	0.20	0.69	0.62	0.79
b_est	0.70	1.57	1.51	1.53	2.87	1.25	3.98	1.20	0.23	0.78	0.55	0.73

MCSM Test 2:

Sig	1	2	3	5	6	8	13	17	18	20	26	30
a_gen	0.25	1.77	0.96	2.48	1.40	0.09	1.91	1.55	1.86	2.45	1.79	2.59
a_est	0.29	1.76	1.00	2.38	1.35	0.17	1.91	1.42	1.83	2.47	1.80	2.56
b_gen	1.55	0.35	3.09	0.13	1.79	3.27	0.55	2.53	1.13	2.29	1.19	0.83
b_est	1.47	0.36	3.32	0.28	1.82	3.43	0.56	2.41	1.22	2.42	1.12	0.80

MCSM Test 3:

Sig	1	2	3	5	6	8	13	17	18	20	26	30
a_gen	0.23	0.37	2.93	3.78	1.01	15.8	1.83	0.27	0.63	0.54	0.69	2.43
a_est	0.20	0.41	2.80	3.39	1.00	15.8	1.73	0.24	0.61	0.57	0.69	2.39
b_gen	0.53	0.23	4.87	2.86	1.96	0.83	0.85	0.49	0.51	0.75	4.34	2.60
b_est	0.39	0.23	4.75	3.00	2.17	0.81	0.93	0.45	0.52	0.68	4.43	2.60

MCSM Test 4

Sig	1	2	3	5	6	8	13	17	18	20	26	30
a_gen	1.34	0.22	0.35	1.78	0.31	0.40	1.44	3.02	3.05	0.39	0.38	0.55
a_est	1.41	0.22	0.36	1.65	0.26	0.43	1.51	3.05	3.10	0.32	0.45	0.55
b_gen	1.05	0.97	0.83	4.80	4.33	1.40	1.43	0.16	0.50	0.35	1.51	6.66
b_est	1.12	0.91	0.86	4.88	4.35	1.28	1.44	0.17	0.51	0.50	1.43	6.84

MCSM Test 5

Sig	1	2	3	5	6	8	13	17	18	20	26	30
a_gen	0.80	1.04	0.10	1.25	0.41	0.20	0.26	1.17	1.51	0.48	0.42	2.32
a_est	0.79	1.04	0.19	1.31	0.43	0.12	0.25	1.18	1.52	0.47	0.34	2.25
b_gen	2.74	1.88	1.38	0.66	0.72	0.27	0.22	1.21	0.96	1.98	0.26	2.76
b_est	2.52	1.88	1.33	0.56	0.72	0.26	0.19	1.08	0.95	1.97	0.22	2.49

First, it can be seen that we are able to estimate the Dirichlet parameters accurately. We also report the average approximation error over all tests to show how accurately the code can reconstruct the parameters. The average approximation error is 9%. For comparison, the average approximation error of a random estimator that estimates the parameters using the same generation method described above is 228%.

Generating data for JMCSM is a little trickier. Given a database, we first learned the inherent exposures in order to use them in the generative process as inherent exposures per sample. Then, we drew Dirichlet parameters, multiplied them by $G = 1000$ and used the product as Dirichlet parameters in the generative process. Note that this is necessary in the JMCSM framework, as it is assumed that each strand's exposure is close to the inherent exposure. Another issue with learning JMCSM is that since we learn the strand bias per signature, we won't necessarily reconstruct the exact parameters. However, the ratio between the normalized parameters $r = \frac{a_i}{\sum_{k=1}^{K} a_k} / \frac{b_i}{\sum_{k=1}^{K} b_k}$ (used in figure 5) should be preserved instead.

To demonstrate the ability of our JMCSM code to reconstruct the ratios, we followed the process described above, and generated 5 independent databases based on BRCA.

The generated ratios and their estimations are reported below:

Sig	1	2	3	5	6	8	13	17	18	20	26	30
r_gen	0.27	2.36	3.10	0.89	6.08	3.06	0.49	0.71	0.68	1.94	0.16	0.68
r_est	0.29	2.46	2.22	0.90	5.15	2.51	0.54	0.74	0.78	1.99	0.26	0.68

Sig	1	2	3	5	6	8	13	17	18	20	26	30
r_gen	2.67	0.76	1.43	1.43	20.3	0.38	0.14	0.53	0.98	1.61	0.60	3.70
r_est	2.36	0.84	1.31	1.46	9.15	0.43	0.16	0.66	0.89	1.63	0.62	2.20

Sig	1	2	3	5	6	8	13	17	18	20	26	30
r_gen	0.56	0.46	0.45	0.54	3.25	0.57	5.41	1.18	1.97	1.22	0.44	0.80
r_est	0.67	0.54	0.56	0.68	2.62	0.71	6.21	1.23	1.59	1.24	0.53	0.93

Sig	1	2	3	5	6	8	13	17	18	20	26	30
r_gen	0.36	1.95	0.04	0.11	0.45	0.05	0.86	6.52	4.27	2.85	0.31	5.68
r_est	0.76	3.94	0.15	0.21	0.97	0.15	1.97	10.1	2.04	2.61	0.69	6.55

Sig	1	2	3	5	6	8	13	17	18	20	26	30
r_gen	1.42	0.07	1.48	0.21	9.39	6.83	1.44	0.65	0.17	0.33	0.39	1.82
r_est	1.68	0.13	1.38	0.31	8.22	5.16	1.97	0.86	0.42	0.70	0.54	1.55

Again, we observe that the algorithm can estimate the ratios (although slightly less accurately than its MCSM variant). We also report the average approximation error over all tests to show how accurately the code can

reconstruct the ratios. The average approximation error is 40%. For comparison, the average approximation error of a random estimator that estimates the parameters using the same generation method described above is 630%.

The authors mention that the Watson/Crick strand information did not lead to good results, but show no data supporting this. They should support this statement with data.

We added the results as Supplementary Table 1.

Reviewer 3

This paper introduces several probabilistic models and corresponding inference algorithms for inferring mutational signature exposures. These models are variations of the Latent Dirichlet Allocation model, capturing covariates among mutational signatures. This paper has been reviewed and accepted at RECOMB-CCB previously. Although I did not review this paper, a point-by-point response is missing, and the paper is still a bit rough (models are hard to read, typos, excessive number of acronyms, etc.). Moreover, the experimental evaluation is not great, lacking a compelling biological use case.

A point-by-point response was included in the original PLOS submission file. We will address the following issues separately.

1. Improve description of the methods

I found the methods hard to read due to undefined variables, distributions and missing intuition. I will list some examples below.

- "The per-sample mutation category count of the two possible feature values are denoted I_{t,m} and J_{t,m}, respectively."

(1) What is m? -- from context one can guess it is a mutation category, but it would be good to be explicit about it.

We have clarified the methods and added all missing definitions (p. 03).

(2) What is a single binary feature? What are the two possible feature values? Why only 2? Motivation is needed here.

We now explain more clearly that a binary feature is the simplest scenario and motivate by the example we analyze of a strand-identity feature (p. 02)

- Why are a_k' ~ Norm(0, sigma^2)? This allows for negative values, whereas Dirichlet concentration parameters must be positive. I have seen gamma distributions being used as a prior for a_k.

Correct. However, we use exp(a_k') as Dirichlet parameters to avoid negative values. We used this method following the paper "Topic Models Conditioned on Arbitrary Features with Dirichlet-multinomial Regression".

- Figure 1 and Section 2.1. gamma is undefined -- do these describe signatures? What are the dimensions?

It is described early in the Preliminaries section: "the signatures are modeled as multinomial distributions, such that the probability to draw a mutation m from signature k is notated by $\gamma_{k,m}$"

- Section 2.1: What is a signature count -- variable N_{t,k}?

The number of times signature k occurs in sample t. We now clarify its definition in the text (p. 03).

- Figure 1: What are variables z and m?

Z indicates the signature category of the current mutation, and m indicates its mutation category. We now clarify their definition in the text (p. 03).

- Figure 1: it would be good to include the conditional distribution for each node (with in-degree > 0). E.g. a_k' ~ N(0, sigma^2).

We added this information as requested (for all relevant figures).

As a general comment, it would help to include a table, comparing the different models. Also, some additional motivation for each model would be welcome.

We clarified the motivation for each model and summarized them in a table as requested.

2. Experimental evaluation

My main concern with the current experimental evaluation is that the case for more complex models that support covariates has not been made. The experimental evaluation does not compare against a baseline method that does not include any covariates (this can be MMM, or just straight up NMF with the signature matrix fixed). Do more complex models allow for better inference with fewer mutations? The paper needs to make this case.

Both models (MCSM and JMCSM) are compared to covariate invariant model (LDA and gLDA, respectively). We clarify it in the text (p. 8).

- I find it interesting that the "[paper] could detect no advantage of the models over their strand oblivious counterparts". Is this because the inference is more challenging for the more complex models? To assess this, simulations with known ground truth would be useful. Do the given algorithms for the more complex models have convergence issues?

See answers to comments 7 and 8 of Reviewer #2.

- Equation (6) lists the empirical log-likelihood of the MCSM model. What about the other models?

The Model Evaluation define the process in which the empirical log-likelihood is calculated, without regard to a specific model.

- Do models with more parameters achieve higher empirical likelihoods by default?

There is no such bias since we compare log-likelihood on held-out data.

Minor

* Figure 5, which model is this? MCSM or JMCSM?

JMCSM. We clarified the text (p. 9)

* Figure 6 is not clear. Include some equations in the main text.

We clarified the test procedure as requested (p. 10).
* Citation needed: "Signatures 2, 13, 26 are known to have a strong replication strand bias"

We added the required citation (p. 9).

* dirichlet => Dirichlet

Fixed.

* signature => Signature

Fixed.
