# Supplementary material for: A mutation-level covariate model for mutational signatures
Source: PLoS Comput Biol. 2023 Jun 5;19(6):e1011195. doi: 10.1371/journal.pcbi.1011195 (PMC10270581; doi:10.1371/journal.pcbi.1011195)
Supplement: Attachment — Submitted filename: 3rd Review Answers V5.pdf [file pcbi.1011195.s003.pdf]

We provide here a point-to-point response the last comments of the reviewers, and indicate whether any changes were made to the paper

Reviewer #1: I had a very hard time linking the authors' response in the point-2-point reply to the changes made in the paper. I did not see any changes highlighted and often failed to identify any improvements. Here are some specific examples:

Authors: "The paper presents novel models that take mutation-level covariates into account, we have now emphasized the novel aspects in the Introduction."
However, advantage over eg [17] is not specified

The model developed in [17], TCSM, considers tumor-level covariates, as opposed to our models that considers mutation-level covariates. We explicitly explain this difference in the Introduction.

Last review: "The link to the covariates is done on parameter α′, i.e. the log-transformed Dirichlet parameter, and as such it determines both mean and variance of signature exposures, which should be made clearer."
Authors: "We have now clarified the way the covariate affect the model (p. 4)."
However, p4 does not make any reference to α′

Some of the pages on which the changes are given are disordered, since we've been asked to re-order the paragraphs after the resubmission. This specific change is given in page 8 in the current version*: "In this model, the binary feature denoted by x determines the parameters of the Dirichlet distribution from which the exposures are drawn"*

Last review: "The interpretation of results is on the normalized Dirichlet parameter α/sum α_i, and is compositional data. Therefore, signature-specific results are difficult and this needs to be made clearer in Figures 5 and 6."
Authors: "We have now explained more clearly the caveats of the parameter ratio approach in Figure 5 and how it is addressed in the results of Figure 6 to pinpoint the relevant biased signatures (p. 10)."
However, p10 does not make any reference to interpretation of parameters

We apologize for the wrong page number – we address this issue explicitly on page 5.

Last review: "The paper the authors claim to present a way of extracting signatures and exposures (e.g. Conclusions: We have developed new topic models to account for mutation-level covariates when learning mutational signatures and their exposures) However, they assume that mutational signatures are known and only estimate the exposures - which needs to be made clearer throughout the paper."
Authors: "We have stated this point (of known signatures) more clearly (p. 3,11)." I could find this change. However, it needs to be clarified if the authors mean “known signatures” by “inherent exposures”?

By "known signatures" we mean that we don’t learn new signatures, and use COSMIC v2 signatures as given. The inherent exposures are not related to that fact.

Last review: "α′ is not defined in text."
Authors: "We added an explicit definition of the a_i and a' parameters (p. 3)"
However, I could not find it on p. 3

Our answer was indeed misleading - a definition of those parameters was already given in the original version of the paper, so it wasn’t highlighted in the last one. These parameters are explained on p. 7.

These discrepancies between the revised manuscript and the P2P are very surprising and I wonder if I have been sent the correct documents.

Reviewer #2: The authors have responded to most of our comments, but most concerns have not been addressed satisfactorily. Even though they improved the exposition problems we and other reviewers mentioned, the algorithm description is still not clear enough. Additionally, we still have doubts about the convergence assessment and the simulation results. We outline these concerns below.

The authors have provided us (but not the main text) with tables showing the progress in held-out log-likelihood of the different models during the Stochastic EM algorithm in order to show that 50 iterations are enough. However, not only do some runs not converge, as they point out, but they also miss the main point of our comment: the method should not be run with a fixed number of iterations for all data sets, as it is natural that the time to convergence varies for different data sets. Instead, they should run their method for as many iterations as it needs to achieve convergence (within some computational budget), based on the progression of the log-likelihood.

As we show below (in our answer to third comment below that starts with "Thirdly,…"), 50 iterations is a conservative estimate that guarantees that the held-out log likelihood has reached a local maximum (this happens in all cases before the 25th iteration) and allows averaging over noisy estimates of this likelihood. Thus, we always report the mean held-out empirical log-likelihood calculated over the 25 last SEM iterations (of 50 total iterations). We refer to it now on p. 12

The authors have also provided us (but not the main text) with some simulation results. The authors assess the ability of their method to recover the simulated parameters from the simulated data and compare them with a random estimator. Firstly, they should clarify what the “random estimator” is.

The random estimator draws parameters from the actual distribution they were drawn from to generate the data. The generative process is discussed in the paper (see figures 4 and 5 and their descriptions for the plate notations of MCSM and JMCSM, on p. 9 and p. 10).

Secondly, they should show their results in a visual way by using boxplots aggregated across different levels of difficulty in the simulations.

We opted to use radar charts to qualitatively show that the model can reconstruct the simulated biases. (We are not interested in the accurate quartiles of the errors since they are affected by the process we use to generate the data with, which is not necessarily representative of the real data.) See our next response for the actual comparison and charts.

Thirdly, and most importantly, the goal of the simulations is to show the performance of each method to recover the simulated true quantities of interest and compare them. Specifically, if the point is to show that the joint model is able to capture strand specificity, the authors must show that it indeed recovers them better than the alternative baseline.

The following results are summarized in the supplementary information section.

We reran five tests on generated data. The data was generated by the same process as in the previous response:

1. For MCSM we drew Dirichlet parameters (a' and b') from a standard normal distribution with $\sigma = 1$ and $\mu = 0$, and generated the mutation data using the generative process described in the text. We did it with different signatures, and number of samples (and their sizes) with respect to the original databases.
2. For JMCSM we used a similar process, and then we multiplied $a = \exp(a')$ and $b = \exp(b')$ by $G = 1000$. Note that this is necessary in the JMCSM framework, as it is assumed that each strand's exposure is close to the inherent exposure.

Then, the data was split to a train set and a test set. This time we also tried to learn the LDA and gLDA parameters, to compare it with the more complex algorithms we offer.

<u>JMCSM vs gLDA</u>

First, we show the parameters the model reconstructed visually. Since we learn the strand bias per signature, we won't necessarily reconstruct the exact parameters. However, the ratio between the normalized parameters $r_i = \frac{a_i}{\sum_{k=1}^{K} a_k} / \frac{b_i}{\sum_{k=1}^{K} b_k}$, that indicates the bias of the signatures and was used in figure 1, should be preserved instead. The radial axis indicates r_i and the angular axis indicates the signature. The results for all 5 tests are given in the following figure (the values of the generated data and of the estimated parameters using JMCSM are in blue and orange respectively):

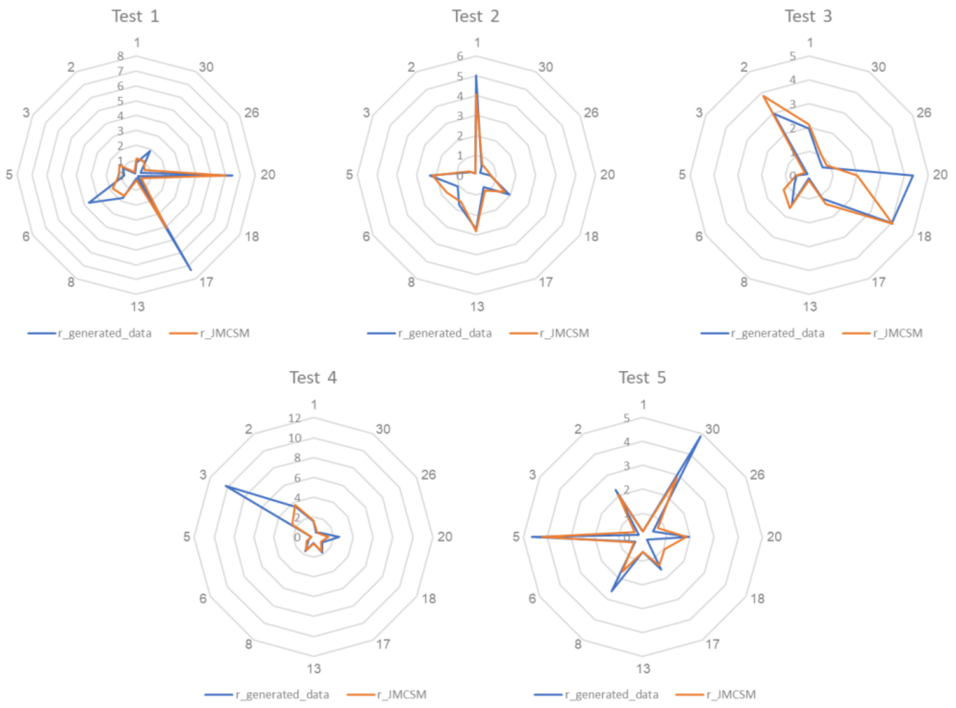

It is evident that JMCSM is able to reconstruct the bias trends in most cases. For example, in test 1 the algorithm recognized that signatures 17 and 20 had the highest biases towards the strand that was drawn with parameters a in test 1, and that signatures 13, 18, 26, 30, have small r_i. However, it didn't calculate the bias of signature 6 correctly. Similar results can be seen in all other tests.

Since gLDA assumes one vector of parameters, equivalently to the assumption that $a_i = b_i$, we would get $r_i = 1$ for every i, which is clearly incorrect.

In the following table we present the held-out log-likelihood calculated using JMCSM and gLDA for each one of the 5 tests we ran.

test no.	ll_JMCSM	ll_gLDA	likelihood diff
1	-1018087	-1022534	-0.435%
2	-1003037	-1008897	-0.581%
3	-1008282	-1016524	-0.811%
4	-974458	-978033	-0.366%
5	-960959	-965160	-0.435%

In all cases, JMCSM dominates gLDA in terms of log-likelihood.

<u>MCSM vs LDA</u>

Here, we can directly compare the reconstruction of the model parameters. We draw similar plots, were the black and the orange dashed lines indicate the a and b parameters of the generated data, the yellow and the green

dashed lines indicate the parameters a and b estimated using MCSM, and the blue line indicates the a parameters estimated using LDA.

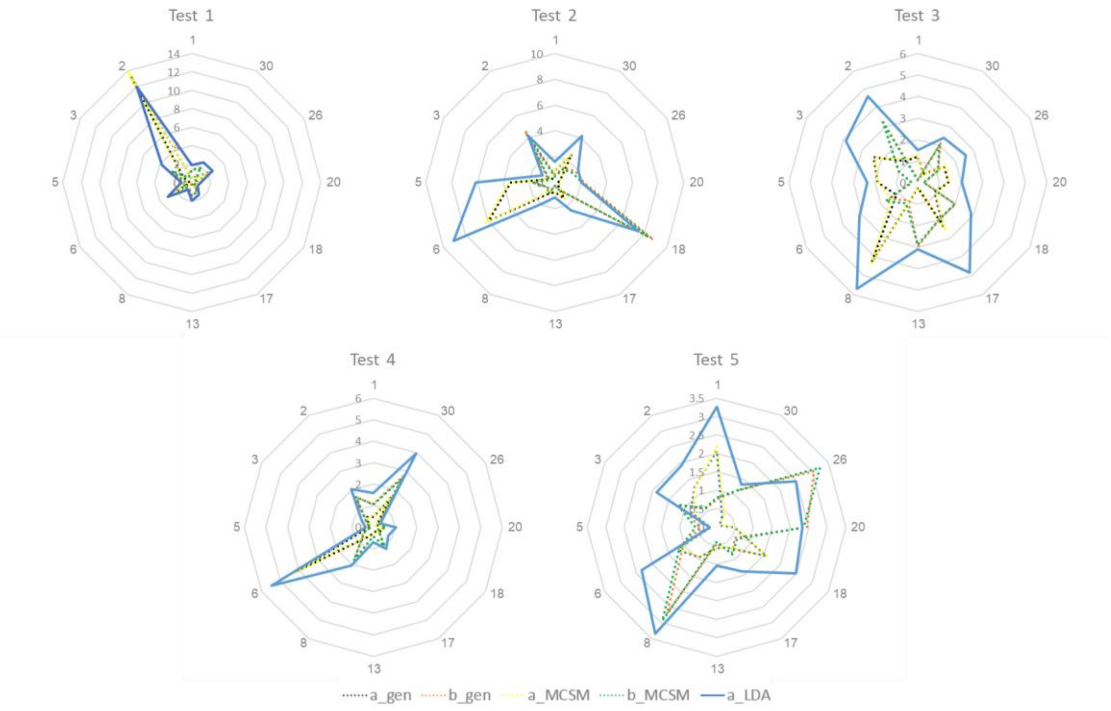

It can be seen that MCSM is able to reconstruct the model parameters well while LDA captures characteristics from both vectors. However, in some cases LDA is good enough to get similar or slightly better held-out log-likelihoods than MCSM (this happened in tests 4 and 5). Note that this coincided with the results in the paper).

Finally, in the following figure we provide the for each iteration (of test 2 for example) the held-out -log(loglikelihood) given the parameters estimated in that iteration for both MCSM and LDA (we couldn't plot the loglikelihoods directly since the difference between the loglikelihood of LDA and MCSM is larger their variance between iterations).

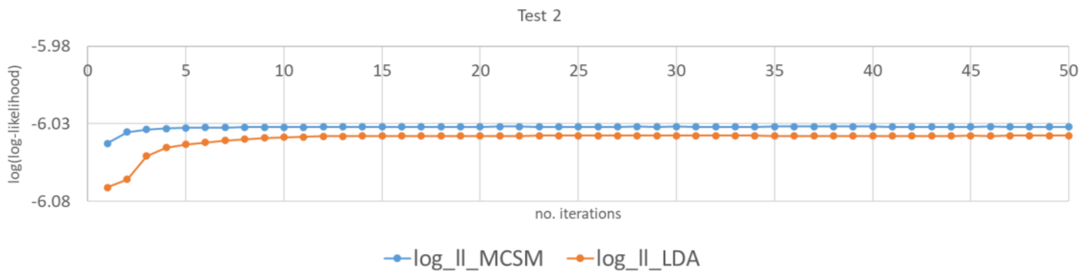

It can be seen that in this case MCSM dominates LDA in every iteration. Also, both algorithms reach a local maximum very fast (after a few iterations), and then stay around it. This effect only happens in MCSM and LDA (and not JMCSM and gLDA) since the Dirichlet parameters have a relatively small concentration parameter, yielding a noisy signature assignment in the stochastic part of the SEM algorithm.

Additionally, just comparing the held-out likelihoods between the different models is not enough to motivate the more complex ones. The question that the authors must answer unequivocally in their paper using simulation and real data results is whether the estimated exposures more closely capture the ground truth using the proposed methods, instead of the simpler alternatives.

We followed the reviewer's request and present the quality of estimated parameters using the simulated data as detailed in the answer above. For real data, ground truth is not known, hence held-out log likelihood is the only applicable measure.

Finally, the code provided in the Github repository is insufficient to reproduce the results in the paper, as it is simply a collection of compressed scripts and does not include instructions on how to run the models on new data.

Clearer instructions are now provided.
