# Supplementary material for: A mutation-level covariate model for mutational signatures
Source: PLoS Comput Biol. 2023 Jun 5;19(6):e1011195. doi: 10.1371/journal.pcbi.1011195 (PMC10270581; doi:10.1371/journal.pcbi.1011195)
Supplement: Attachment — Submitted filename: Response.docx [file pcbi.1011195.s004.docx]

Unfortunately, the authors still seem to miss our point about assessing their methods’ ability to obtain the true parameters from simulated data. In order to illustrate the advantages of the strand-specific models, the authors must simulate data sets with different levels of strand-specific effects, and assess the ability of each method to recover the simulated exposures. They must then report some metric that summarizes this performance across many instances of each simulation case, instead of plotting the learned parameter values on top of the true values for each instance of the simulation. For example, they may report the error between the true versus estimated exposures and show the distributions of these errors for each method, and each simulation setting across all replicates.

We opted to use radar charts (as were given in the latest response to the reviewers) to qualitatively show that the model can reconstruct the simulated parameters and biases. We believe that error statistics calculated using generated data are of less interest, as these would be affected by the process used to generate the data, which is not necessarily representative of real data.

Additionally, while the authors did improve their simulation study, its description is still very sparse and not reproducible. The authors must be clear and detailed about their procedures for simulating and evaluating the methods on those simulations. For example, “Then, the data was split to a train set and a test set.” is not enough. Please provide the actual splitting procedure.

This quote is from our previous response but in the paper itself we do provide a detailed explanation on the splitting procedure.

They also explained in their rebuttal what the “random estimator” is, but did not add that information to the manuscript. And the small simulation results they show in the review are still not added to the main text.

The "random estimator" is again quoted from our previous response but is not mentioned in the manuscript. Per the reviewer request we added the simulation results to the supplement with the caveat that they may not be representative of real data.

Finally, while we do agree that the plot indicates that convergence is reached well before 50 iterations in that particular case, there is no reason to believe that this will generalise to other datasets. We advise to define a more general convergence criterion as is commonly done.

Following the reviewer's comment, we now provide a convergence criterion (achieving the local held-out log-likelihood maximum) and show that it is satisfied by our 50 iteration choice.

Reviewer #2: **No:** Code for simulation is not available; thus not all results are reproducible.

A link to the code in GitHub is given in the manuscript and it includes the code that was used to test our methods on simulated data (we also refer to it in out code instruction .docx file).
